# Management and Treatment of Concussions via Tele-Concussion in a Pediatric Setting: Methodological Approach and Descriptive Analysis

**DOI:** 10.2196/19924

**Published:** 2020-08-13

**Authors:** Todd Caze II, Gregory P Knell, John Abt, Scott O Burkhart

**Affiliations:** 1 Children's Health Andrew's Insitute Plano, TX United States; 2 University of Texas-Southwestern Medical Center Dallas, TX United States; 3 Department of Epidemiology, Human Genetics, and Environmental Sciences The University of Texas Health Science Center at Houston Houston, TX United States; 4 Center for Pediatric Population Health The University of Texas Health Science Center at Houston Houston, TX United States

**Keywords:** brain concussion, athletic injuries, sports injuries, telemedicine, eHealth, mHealth, telehealth, mobile health, adolescent, child, COVID-19

## Abstract

**Background:**

Approximately 2 million children in the United States sustain a concussion annually, resulting in an economic impact as high as US $20 billion. Patients who receive treatment at concussion specialty clinics, versus primary care, experience faster recovery, thereby reducing patient burden and subsequent medical-related costs. Accessibility to specialty clinics is typically limited by the availability of in-office visits. This is particularly relevant in light of the severe acute respiratory syndrome coronavirus 2 pandemic and subsequent guidance to eliminate all non–medically necessary in-clinic visits. Telehealth has been used to effectively deliver in-clinic care across several disciplines including psychiatry, psychology, and neuropsychology. However, a model of telehealth delivered concussion assessment, treatment, and management has not been established.

**Objective:**

The purposes of this paper are to describe a pediatric concussion specialty clinic’s experiences in delivering telehealth concussion services and to provide preliminary descriptive data on a sample of pediatric telehealth patients with concussions.

**Methods:**

The specialty pediatric concussion clinic described here began providing telehealth services in 2019 and is part of the largest and fastest-growing telehealth hospital network in the United States. The clinical care process will be described, including accessing the telehealth platform, assessment during the initial appointment, injury management including communication with relevant patient stakeholders (eg, parent or guardians, athletic trainers), dissemination of rehabilitation exercises, and nature of follow-up visits. Descriptive data will include patient demographics, the radius of care, the time between the date of injury and initial visit, the average number of follow-up visits, and days until medically cleared for return-to-learn and return-to-play.

**Results:**

The analytic sample included 18 patients with concussions who were seen for all of their visits via telehealth between August 2019 and April 2020. The mean age of the sample was 14.5 (SD 2.5) years. The radius of care was a median of 17 (IQR 11.0-31.0) miles from the clinic with a median time between injury and the first visit of 21 (IQR 6.0-41.5) days. The mean number of visits was 2.2 (SD 0.8) with a median days between visits of 5.4 (IQR 3.0-9.3) to manage and treat the concussion. Of the 18 patients, 55.6% (n=10) were medically cleared for return-to-learn or -play in a median of 15.5 (IQR 11.0-29.0) days.

**Conclusions:**

Limited access to health care is a well-understood barrier for receiving quality care. Subsequently, there are increasing demands for flexibility in delivering concussion services remotely and in-clinic. This is the first paper to provide a clinically relevant framework for the assessment, management, and treatment of acute concussion via telehealth in a pediatric population.

## Introduction

### Background

Sport-related concussions (SRCs) are a significant public health concern in the United States [1]. The prevalence rate of SRCs among US children (<18 years) is approximately 1400-2400 per 100,000 children, amounting to 1.9 million annual cases [2]. Pediatric SRCs increase the risk of short-term health problems including neurobehavioral changes (eg, fatigue, nervousness or irritability), cognitive impairment (eg, slowed reaction times, difficultly in concentrating), sleep disturbances, somatic symptoms (eg, nausea, vomiting, dizziness), or emotional symptoms [3]. Long-term problems include psychosocial outcomes (eg, hyperactivity, inattention) [4] during later childhood years and psychiatric disorders and premature mortality in adulthood [5]. These poor health outcomes related to concussions lead to a staggering economic impact on society [6]. There is evidence, however, that early diagnosis and treatment decrease symptom severity and reduce recovery time, diminishing the risk of short- and possibly long-term health outcomes [4,7].

### Current Status

Recent advancements in the understanding of concussion management have resulted in better outcomes for patients. Beginning in 2001 the Concussion in Sport Group began releasing international consensus statements with recommended guidelines for the identification and management of concussions [8]. Over the past four consensus statements, return-to-learn (RTL) and return-to-play (RTP) guidelines have become more prescriptive and step-wise. RTL currently includes systematic progression from minimum academic activity with accommodations to a gradual increase in performing all academic activities equivalent to before injury [9]. RTP currently includes systematic progression from minimum physical activity to eventual full-return of sport-specific activity, including full-contact practice [9]. This step-wise progression for RTP has resulted in an increase in average days of recovery in high school and collegiate athletes, as well as a significant reduction in the number of repeated concussions and duration between multiple concussions [10]. Additionally, international and national consensus statements advocate for targeted referrals to specialty clinics (eg, physical, vestibular, or cervical therapy). These specialty clinics then hold the responsibility to determine RTP and RTL [10-12]. Accordingly, there has been an increase in referrals to specialty multidisciplinary concussion clinics based on the evolution of concussion consensus statements and guidelines [13].

### Challenges

Despite an increased need for specialty concussion clinics, access is limited. It is well documented [14] that the physical distance between patients and clinics limits patient accessibility. This assumes the patient has access to a reliable mode of personal or public transportation. Given that concussion specialty clinics are relatively rare in the United States and typically only located in major metropolitan areas [15,16], access for the majority of US residents including those residing in rural areas is particularly limited. The scarcity of specialty concussion clinics further limits the availability of appointments and makes it difficult to receive appropriate care in the acute phase of injury [17,18]. This is important because early symptom burden is often the strongest predictor of recovery [19], with targeted interventions being more effective than rest and graded-exertion alone [20].

A possible mechanism to overcome the issue of accessibility is remotely delivered care or telehealth. Telehealth platforms have been used to effectively deliver in-clinic care across several disciplines including primary care, neurology, behavioral health, psychiatry, and neuropsychology [21-25]. Research shows no difference in patient ratings of therapeutic alliance and treatment satisfaction of in-clinic visits versus telehealth platforms, including concussion care and management delivered through telehealth (tele-concussion) specifically [26]. Despite some preliminary evidence on the efficacy of tele-concussion, few providers offer these services. However, the severe acute respiratory syndrome coronavirus 2 (SARS-CoV-2) outbreak in March 2020 forced many providers to offer telehealth services to slow transmission by limiting person-to-person exposures [27]. This recent rise in tele-services and general overall client satisfaction will likely lend itself to a further increase in use of telemedicine [28].

### Solutions

However, best practices for telehealth delivery for the assessment, treatment, and management of concussions have not been established. The primary purpose of this paper is to describe the methodological approaches of one pediatric concussion clinic’s transition to tele-concussion. We will also provide preliminary descriptive data on a sample of pediatric telehealth patients with concussions. Providing an example of how one clinic implements tele-concussion services can spur other providers to work through concerns and implementation logistics. In addition, preliminary descriptive data can help the field in discerning what are important variables to consider in future empirical validation studies on tele-concussion.

## Methods of Service Delivery

### Children’s Health Andrews Institute

Children’s Health Andrews Institute (CHAI) is a pediatric sports medicine and orthopedic clinic located in the Dallas/Fort Worth metropolitan area. Within CHAI, there are several specialty clinics including a concussion clinic. This multidisciplinary concussion clinic saw over 600 new pediatric concussions in 2019 with a portion of those seen via telehealth for follow-up visits only. Following the SARS-CoV-2 outbreak, CHAI began a transition to an all telehealth setting for all patient visits in March 2020. To have an understanding of the clinic’s methodological approach to tele-concussion, we will provide a brief overview of the in-person approach.

### In-Person Clinic Methods

Consistent with consensus guidelines [9], once a patient is suspected of sustaining a concussion, they are evaluated in-clinic and provided targeted recommendations for RTL and RTP. This initial evaluation includes a brief clinical interview along with symptom, vestibular, ocular motor, and cognitive screening. Once concussion diagnosis is confirmed, the patient is given paperwork outlining individualized RTL and RTP progressions that the patient disseminates to necessary school personnel. Outside specialty referrals are made as necessary (eg, physical therapy, vestibular therapy) and follow-up visits scheduled.

During the follow-up visit, progress and recovery are re-evaluated through a brief clinical interview; symptom screening; and vestibular, ocular motor, and cognitive functioning screening. This aggregate data informs modifications to recovery protocols for RTL and RTP. If recovery is not progressing or symptoms are worsening, a referral is made (eg, physical therapy). Depending on progression of RTL and RTP protocols, patients may require additional follow-up visits.

At a patient’s clearance and final visit, the treating provider determines if the patient has successfully completed RTL and RTP protocols. During the final visit, a clinical interview; symptom endorsement; and vestibular, ocular motor, and cognitive functioning screening are conducted to determine if the patient has returned to their baseline levels. Once cleared by treatment provider, the patient is provided a medical clearance note for school and sports. Although the total number of visits vary, the average is 2-3 visits spanning over several weeks.

### Tele-Concussion Clinic Methods

The first step in transitioning an in-clinic concussion management program to a telehealth delivered program is to establish a video delivery platform accessible to patient and provider. There are various telehealth platforms that can serve as this communication tool. It is important to discuss with your organization which platforms are compliant with their institutional policies and standards such as the Health Insurance Portability and Accountability Act (HIPPA). The CHAI concussion clinic uses an internal entity, which patients can access via a website or mobile app (requires downloading to a mobile device), though other HIPPA compliant telehealth videoconferencing platforms can be used.

The second step that should be considered in transitioning an in-clinic concussion management program to a telehealth delivered program is to convert all patient materials that are typically delivered or provided to the patient in hard copies into digital copies. At CHAI, this includes a patient handout describing what a concussion is and general tips to help maximize recovery. These materials are delivered to patients at CHAI through an internal patient portal, but they could also be delivered via email or other patient portals if available through a hospital network.

The third step in this transition is to convert all testing delivered to the patient in-clinic to a virtual delivery. In the CHAI concussion clinic, this included the Post-Concussive Symptom Checklist (PCSS) [29], the Vestibular Ocular Motor Screener (VOMS) [30], and computerized neurocognitive testing. The PCSS is a self-reported symptom checklist that was converted to a digital form that can be delivered to the patient via email or patient portal. If administered on a digital platform (eg, RedCap, My Patient Portal), the patient can receive, complete, and return the form online. Otherwise, the patient may have to print the document, complete it, and scan or return it to the provider. In the CHAI concussion clinic, patients fill out the PCSS online via patient portal at the beginning of their virtual visit. For the VOMS, there are several steps necessary to administer this screening tool virtually. First, patients are informed prior to their visit that the following materials are needed: a ruler with centimeters, a pencil, and a metronome (this can be through a mobile phone app or online). Prior to administration of the VOMS, patients are instructed to have their cameras positioned so the clinician can see their eye movements during the screening. Second, similar to in-person administration, a baseline for symptoms (Likert-scale 0-10) of headache, dizziness, nausea, and fatigue are collected. After a baseline of symptoms are collected, in-person administration of the VOMS requires the clinician to provide the stimulus to guide the patient in performing the vestibular and ocular movements of the screening while noting if any of these movement patterns provoke symptoms (an increase from baseline symptoms collected). For telehealth, the clinician is still guiding the patient through movements, but they are now directing the patient on how they can do these movements on their own. This requires the clinician to first demonstrate how to use a pencil to perform the movements of the ocular screening portion of the VOMS: smooth pursuits, vertical and horizontal saccades, and convergence with the additional use of a ruler in centimeters. Each portion of the ocular motor screening is demonstrated by the clinician and then practiced by the patient to ensure accuracy. Once the patient is able to perform a specific portion of the ocular screening, such as smooth pursuits, the patient then rerates symptoms of headache, dizziness, nausea, and fatigue to note if that movement pattern resulted in symptom provocation. The clinician then demonstrates with pencil and metronome how the patient is to perform movements of the vestibular screening of the VOMS: horizontal and vertical vestibular-ocular reflex, and visual motion sensitivity test. Similar to the ocular motor section of the VOMS, each portion of the vestibular screening is demonstrated by the clinician and then practiced by the patient to ensure accuracy. Once the patient is able to perform a specific portion of the vestibular screening, such as vestibular-ocular reflex, the patient then rerates symptoms of headache, dizziness, nausea, and fatigue to note if that movement pattern resulted in symptom provocation. For computerized neurocognitive testing, similar to in-person visits, supervision of testing is provided during telehealth visits.

The fourth step in this process is to prepare any other materials for a postclinic visit that may be needed to supplement care. Similar to in-person visits, individualized paperwork outlining RTL and RTP are provided to the patient but through the patient portal. This paperwork is then disseminated to necessary school personnel by the patient.

Finally, the actual patient visit requires some steps to successfully transition an in-clinic concussion management program to a telehealth delivered program. Although patient clinical care in the tele-concussion clinic is similar to the in-person clinic, an important difference is the previsit preparation. Before the initial visit or assessment the patient’s parents or guardians are emailed instructions on how to access the virtual health platform. Patients are instructed to log into the telehealth app prior (approximately 15 minutes) to all appointments to troubleshoot any technical difficulties that may arise. A member of the concussion clinical team is made available to the patient via telehealth, if necessary, to assist with any problems connecting. All other elements of the tele-concussion visits remain the same as the in-person (referrals to specialty clinics, etc).

### Preliminary Data

A secondary objective of this paper is to provide preliminary descriptive data on a sample of pediatric telehealth patients with concussions. Descriptive statistics on patient demographics (age, sex, race or ethnicity, and patient location), patient visits (time to first visit, total number of visits, time between visits), and patient recovery (recovery time, medical clearance) were evaluated for missingness and normality, and reported appropriately as proportions, means, medians, and ranges.

All data were collected from a deidentified clinical database and received exempt status from the Children’s Health Institutional Review Board.

## Evaluation Outcomes

Participants’ (N=18) age ranged from 9-20 years, with a mean of 14.5 (SD 2.5) years. The majority of patients (n=14, 77.8%) were female, non-Hispanic White (n=14, 77.8%), located in-state (n=17, 94.4%), and medically cleared for RTP or RTL (n=10, 56.6%). Participants were located in a median of 17 (IQR 11.0-31.0) miles from the CHAI and required a mean of 2.2 (SD 0.8) visits. There was a median of 5.4 (IQR 3.0-9.3) days between visits, and patients took a median of 15.5 (IQR 11.0-29.0) days to recover fully (see Table 1 for more details).

**Table 1:** Descriptive statistics on patients using a pediatric tele-concussion clinic, 2019-2020 (N=18).

Characteristic		Value
Age (years), mean (SD)		14.5 (2.5)
**Sex, n (%)**		
	Male	4 (22.2)
	Female	14 (77.8)
**Race/ethnicity, n (%)**		
	Non-Hispanic White	14 (77.8)
	Hispanic	3 (16.7)
	Non-Hispanic Black	1 (5.6)
**Patient location**		
	In state, n (%)	17 (94.4)
	Distance from clinic (miles), median (IQR)	17 (11.0-31.0)
	Distance from clinic (miles), range	2-863
**Patient visits**		
	Time to first visit (days), median (IQR)	21 (6.0-41.5)
	Time between visits (days), median (IQR)	5.4 (3.0-9.3)
	Total number of visits, mean (SD)	2.2 (0.8)
	Total number of visits, range	1-4
**Patient recovery**		
	Recovery time (days), median (IQR)	15.5 (11.0-29.0)
	Medically cleared, n (%)	10 (55.6)

## Discussion

### Telehealth for Concussion Care

In the wake of the SARS-CoV-2 outbreak, general medicine and specialty clinics are rapidly adapting delivery of clinical care. Consistent with the primary objective of this paper, the methodology of converting a specialty clinic’s in-person visit to a telehealth visit were described. Telehealth for concussion care was initially implemented at this clinic starting in 2019. This previous experience in telehealth delivery allowed for an easier transition to all telehealth visits when mandated by the hospital system on March 17, 2020, in response to the SARS-CoV-2 outbreak.

### Potential Challenges of Telemedicine

This study demonstrates how to administer tele-concussion services effectively. However, this does not come without challenges. First, adapting administration of measures such as VOMS to telehealth took time and practice. At CHAI concussion clinic, providers practiced telehealth administration multiple times with one another to ensure familiarity and to troubleshoot administrative difficulties. Clinics will have to consider similar preliminary exercises for any measures delivered via telehealth.

Second, technical issues were unavoidable. Internet bandwidth and Wi-Fi quality varies for patients and can result in connectivity issues. In our experiences, having the patient log-in to their visit prior to the appointment provides opportunities for troubleshooting including log-in assistance. This also provides opportunities to remind patients to minimize distractions, use a larger screen (ie, computer or tablet), and maximize internet speed (ie, have others log-off shared Wi-Fi, using a phone’s Wi-Fi hotspot for the device used for the visit). Other technical difficulties will inevitably arise and keeping a log of issues with resolutions as a quick reference will prove valuable. Additionally, providing the patient with a one-page document, in simple language, with visual instructions on how to access the telehealth platform and information on materials needed for the visit, such as items to assist with VOMS administration, is useful. Some telehealth platforms, including the one used in this study, may automatically disseminate this information. Although most technical difficulties can be resolved through troubleshooting, there are times a visit will simply have to be rescheduled.

Finally, outside referrals may need to be made in areas beyond your typical referral network radius, requiring a widening of known specialty providers such as physical or vestibular therapy. In rare instances, an outside referral may require the patient to travel. It is important to highlight that patients from rural areas are often accustomed to occasionally traveling longer distances for specialty care.

### Future Research

The results presented herein found the average recovery time was within the expected 28-day window [19], and the number of average visits was consistent with the number of in-person clinic visits. These preliminary data demonstrate the potential utility of tele-concussion services; however, future research is needed to expand upon and validate these findings. Future studies should also include examining potential differences in access to clinic and average recovery time of tele-concussion versus in-person clinic treatment. The measures delivered via telehealth should also be validated before being widely disseminated.

### Conclusions

The recent SARS-CoV-2 pandemic created unprecedented alterations to the delivery of medical care. As such, there has been an increase in specialty clinic providers transitioning treatment platforms to telemedicine. Despite the unfortunate circumstances, patient accessibility to specialty concussion clinics will increase, thus providing the opportunity for a reduction in health care costs associated with concussion management [31]. This paper highlights how one clinic transitioned to tele-concussion delivery, providing considerations for how others might embark on a similar transition. Though initial adaption to technology will present challenges, this paper presented some suggestions to facilitate the transition to telehealth. Preliminary data shows promise that the average number of visits and average recovery time are comparable to in-person clinic treatment, with future research needed to confirm these findings. To our knowledge, this is the first paper to provide a clinically relevant framework for the assessment, management, and treatment of acute concussion via telehealth in a pediatric population.

## Abbreviations

CHAI: Children's Health Andrews Institute
HIPPA: Health Insurance Portability and Accountability Act
PCSS: Post-Concussive Symptom Checklist
RTL: return-to-learn
RTP: return-to-play
SARS-CoV-2: severe acute respiratory syndrome coronavirus 2
SRC: sport-related concussions
VOMS: Vestibular Ocular Motor Screener

## References

[1] Rivara FP, Graham R (2014). Sports-related concussions in youth: report from the Institute of Medicine and National Research Council. JAMA.

[2] Bryan MA, Rowhani-Rahbar A, Comstock RD, Rivara F, Seattle Sports Concussion Research Collaborative (2016). Sports- and recreation-related concussions in US youth. Pediatrics.

[3] Willer B, Leddy JJ (2006). Management of concussion and post-concussion syndrome. Curr Treat Options Neurol.

[4] McKinlay A, Dalrymple-Alford JC, Horwood LJ, Fergusson DM (2002). Long term psychosocial outcomes after mild head injury in early childhood. J Neurol Neurosurg Psychiatry.

[5] Sariaslan A, Sharp DJ, D'Onofrio BM, Larsson H, Fazel S (2016). Long-term outcomes associated with traumatic brain injury in childhood and adolescence: a nationwide Swedish cohort study of a wide range of medical and social outcomes. PLoS Med.

[6] Humphreys I, Wood RL, Phillips CJ, Macey S (2013). The costs of traumatic brain injury: a literature review. Clinicoecon Outcomes Res.

[7] Iverson GL, Gioia GA (2016). Returning to school following sport-related concussion. Phys Med Rehabil Clin N Am.

[8] Aubry M, Cantu R, Dvorak J, Graf-Baumann T, Johnston K, Kelly J, Lovell M, McCrory P, Meeuwisse W, Schamasch P, Concussion in Sport Group (2002). Summary and agreement statement of the First International Conference on Concussion in Sport, Vienna 2001. Recommendations for the improvement of safety and health of athletes who may suffer concussive injuries. Br J Sports Med.

[9] McCrory P, Meeuwisse W, Dvořák Jiří, Aubry M, Bailes J, Broglio S, Cantu RC, Cassidy D, Echemendia RJ, Castellani RJ, Davis GA, Ellenbogen R, Emery C, Engebretsen L, Feddermann-Demont N, Giza CC, Guskiewicz KM, Herring S, Iverson GL, Johnston KM, Kissick J, Kutcher J, Leddy JJ, Maddocks D, Makdissi M, Manley GT, McCrea M, Meehan WP, Nagahiro S, Patricios J, Putukian M, Schneider KJ, Sills A, Tator CH, Turner M, Vos PE (2017). Consensus statement on concussion in sport-the 5 international conference on concussion in sport held in Berlin, October 2016. Br J Sports Med.

[10] McCrea M, Broglio S, McAllister T, Zhou W, Zhao S, Katz B, Kudela M, Harezlak J, Nelson L, Meier T, Marshall SW, Guskiewicz KM, CARE Consortium Investigators (2020). Return to play and risk of repeat concussion in collegiate football players: comparative analysis from the NCAA Concussion Study (1999-2001) and CARE Consortium (2014-2017). Br J Sports Med.

[11] Broglio SP, Collins MW, Williams RM, Mucha A, Kontos AP (2015). Current and emerging rehabilitation for concussion: a review of the evidence. Clin Sports Med.

[12] Giza CC, Kutcher JS, Ashwal S, Barth J, Getchius TSD, Gioia GA, Gronseth GS, Guskiewicz K, Mandel S, Manley G, McKeag DB, Thurman DJ, Zafonte R (2013). Summary of evidence-based guideline update: evaluation and management of concussion in sports: report of the Guideline Development Subcommittee of the American Academy of Neurology. Neurology.

[13] Mitchell SH, Hildenbrand K, Pietz K (2016). Emergency physicians' knowledge of sports-related concussion, referral patterns, and use of return to play guidelines. Athletic Training Sports Health Care.

[14] Marmot M, Allen J, Bell R, Bloomer E, Goldblatt P, Consortium for the European Review of Social Determinants of Healththe Health Divide (2012). WHO European review of social determinants of health and the health divide. Lancet.

[15] Rosenthal MB, Zaslavsky A, Newhouse JP (2005). The geographic distribution of physicians revisited. Health Serv Res.

[16] Rosenblatt RA, Andrilla CHA, Catlin M, Larson EH (2015). Geographic and specialty distribution of US physicians trained to treat opioid use disorder. Ann Fam Med.

[17] Cama S, Malowney M, Smith AJB, Spottswood M, Cheng E, Ostrowsky L, Rengifo J, Boyd JW (2017). Availability of outpatient mental health care by pediatricians and child psychiatrists in five U.S. cities. Int J Health Serv.

[18] Miller AR, Armstrong RW, Mâsse LC, Klassen AF, Shen J, O'Donnell ME (2008). Waiting for child developmental and rehabilitation services: an overview of issues and needs. Dev Med Child Neurol.

[19] Iverson GL, Gardner AJ, Terry DP, Ponsford JL, Sills AK, Broshek DK, Solomon GS (2017). Predictors of clinical recovery from concussion: a systematic review. Br J Sports Med.

[20] Schneider KJ, Leddy JJ, Guskiewicz KM, Seifert T, McCrea M, Silverberg ND, Feddermann-Demont N, Iverson GL, Hayden A, Makdissi M (2017). Rest and treatment/rehabilitation following sport-related concussion: a systematic review. Br J Sports Med.

[21] Davis LE, Coleman J, Harnar J, King MK (2014). Teleneurology: successful delivery of chronic neurologic care to 354 patients living remotely in a rural state. Telemed J E Health.

[22] Fortney JC, Pyne JM, Turner EE, Farris KM, Normoyle TM, Avery MD, Hilty DM, Unützer J (2015). Telepsychiatry integration of mental health services into rural primary care settings. Int Rev Psychiatry.

[23] Lindsay JA, Kauth MR, Hudson S, Martin LA, Ramsey DJ, Daily L, Rader J (2015). Implementation of video telehealth to improve access to evidence-based psychotherapy for posttraumatic stress disorder. Telemed J E Health.

[24] Munro Cullum C, Hynan LS, Grosch M, Parikh M, Weiner MF (2014). Teleneuropsychology: evidence for video teleconference-based neuropsychological assessment. J Int Neuropsychol Soc.

[25] Wechsler LR (2015). Advantages and limitations of teleneurology. JAMA Neurol.

[26] McElroy M, Stephenson-Brown K, Mohler S, Elbin RJ (2016). Comparing patient satisfaction between face-to-face and telehealth clinical visits for sport-realted concussion. Int J Exerc Sci: Conference Proc.

[27] Patel PD, Cobb J, Wright D, Turer R, Jordan T, Humphrey A, Kepner AL, Smith G, Rosenbloom ST (2020). Rapid development of telehealth capabilities within pediatric patient portal infrastructure for COVID-19 care: barriers, solutions, results. J Am Med Inform Assoc.

[28] Keshvardoost S, Bahaadinbeigy K, Fatehi F (2020). Role of telehealth in the management of COVID-19: lessons learned from previous SARS, MERS, and Ebola outbreaks. Telemedicine and e-Health.

[29] Lovell MR, Collins MW (1998). Neuropsychological assessment of the college football player. J Head Trauma Rehabil.

[30] Mucha A, Collins MW, Elbin RJ, Furman JM, Troutman-Enseki C, DeWolf RM, Marchetti G, Kontos AP (2014). A Brief Vestibular/Ocular Motor Screening (VOMS) assessment to evaluate concussions: preliminary findings. Am J Sports Med.

[31] Silverberg ND, Gardner AJ, Brubacher JR, Panenka WJ, Li JJ, Iverson GL (2015). Systematic review of multivariable prognostic models for mild traumatic brain injury. J Neurotrauma.

